# Supplementary material for: Coumarin Promotes Hypocotyl Elongation by Increasing the Synthesis of Brassinosteroids in Plants
Source: Int J Mol Sci. 2025 Jan 27;26(3):1092. doi: 10.3390/ijms26031092 (PMC11817769; doi:10.3390/ijms26031092)
Supplement: Supplementary file 1 [file ijms-26-01092-s001.zip › ijms-3393018-supplementary.pdf]

Table S1. The sequeces of genes involved in BRs synthesis or signaling

Names	Sequences
DET2-F:	5' ATTACTTCCACCGCACCA 3'
DET2-R:	5' TTCCGTCTTCGTAGTCATCC 3'
DWF4-F:	5' CCGTACACC GCCACAACAC 3'
DWF4-R:	5' CTACATTCAAAGAGCCTTCC 3'
CPD-F:	5' GACGCTACGAGTGGCTAA 3'
CPD-R:	5' GAACCGCTCTAAACGATG 3'
ROT3-F:	5' ATGCAACCTCCGGCAAGCG 3'
ROT3-R:	5' CGCTTGCCGGAGGTTGCAT 3'
CYP90D1-F:	5' TGCCCTCAATCTCCTAAC 3'
CYP90D1-R:	5' ACATCTTTCATCGCCTTT 3'
BRI1-F:	5' GCGTCCCTTGCTGGTAGT 3'
BRI1-R:	5' CCGCCTCTTTCTTTCTCC 3'
BZR1-F:	5' GCGGTGAACCAAATAACA 3'
BZR1-R:	5' TAGAAGTCGGCGATGAGA 3'
BIN2-F:	5' CACAATGTTGCTGGAGTT 3'
BIN2-R:	5' TGTAAGAAATGTTGGCTTC 3'
BAK1-F:	5' AAAGTAGTGTCGCCAAAT 3'
BAK1-R:	5' GACGCACAAGGATAGAGT 3'
BES1-F:	5' CTCGCCTACCTTCAATCT 3'
BES1-R:	5' ATCCTCCATAGCCACATC 3'
ACTIN2-F:	5' GCTCCTCTTAACCCAAAGGC 3'
ACTIN2-R:	5' CACACCATCACCAGAATCCAGC 3'
